# Exploring the potential of cell-free RNA and Pyramid Scene Parsing Network for early preeclampsia screening

**DOI:** 10.1186/s12884-025-07503-5

**Published:** 2025-04-14

**Authors:** Zhuo Zhao, Xiaoxu Liu, Yonghui Guan, Chunfang Li, Zheng Wang

**Affiliations:** 1 Key Laboratory of Shaanxi Province for Craniofacial Precision Medicine Research, College of Stomatology, Xi’an Jiaotong University, No.98, Xiwu Road, Xi’an, Shaanxi People’s Republic of China; 2 Clinical Research Center of Shaanxi Province for Dental and Maxillofacial Diseases, College of Stomatology, Xi’an Jiaotong University, No.98, Xiwu Road, Xi’an, Shaanxi People’s Republic of China; 3 State Key Laboratory for Manufacturing System Engineering, Xi’an Jiaotong University, Xi’an, China; 4 Department of Physiology and Pathophysiology, School of Basic Medical Sciences, Xian Jiaotong University, Xi’an, China; 5 Department of Urology, The First Affiliated Hospital of Xinjiang Medical University, Urumqi, China; 6 Department of Obstetrics & Gynecology, The First Affiliated Hospital of Xi’an Jiaotong University, No.76, Yanta West Road, Xi’an, Shaanxi People’s Republic of China

**Keywords:** Preeclampsia, Prediction model, Circulating cell-free RNA, Deep learning

## Abstract

**Background:**

Circulating cell-free RNA (cfRNA) is gaining recognition as an effective biomarker for the early detection of preeclampsia (PE). However, the current methods for selecting disease-specific biomarkers are often inefficient and typically one-dimensional.

**Purpose:**

This study introduces a Pyramid Scene Parsing Network (PSPNet) model to predict PE, aiming to improve early risk assessment using cfRNA profiles.

**Methods:**

The theoretical maximum Preeclamptic Risk Index (PRI) of patients clinically diagnosed with PE is defined as “1”, and the control group (NP) is defined as “0”, referred to as the clinical PRI. A data preprocessing algorithm was used to screen relevant cfRNA indicators for PE. The cfRNA expression profiles were obtained from the Gene Expression Omnibus (GSE192902), consisting of 180 normal pregnancies (NP) and 69 preeclamptic (PE) samples, collected at two gestational time points: ≤ 12 weeks and 13–20 weeks. Based on the differences in cfRNA expression profiles, the Calculated Ground Truth values of the NP and PE groups in the sequencing data were acquired (Calculated PRI). The differential algorithm was embedded in the PSPNet neural network and the network was then trained using the generated dataset. Subsequently, the real-world sequencing dataset was used to validate and optimize the network, ultimately outputting the PRI values of the healthy control group and the PE group (PSPNet-based PRI). The model’s predictive ability for PE was evaluated by comparing the fit between Calculated PRI (Calculated Ground Truth) and PSPNet-based PRI.

**Results:**

The mean absolute error (MAE) between the Calculated Ground Truth the PSPNet-based PRI was 0.0178 for cfRNA data sampled at ≤ 12 gws and 0.0195 for data sampled at 13–20 gws. For cfRNA data sequenced at ≤ 12 gws and 13–20 gws, the corresponding loss values, maximum absolute errors, peak-to-valley error values, mean absolute errors, and average prediction times per sample were 0.0178 (0.0195).

**Conclusions:**

The present PSPNet model is reliable and fast for cfRNA-based PE prediction and its PRI output allows for continuous PE risk monitoring, introducing an innovative and effective method for early PE prediction. This model enables timely interventions and better management of pregnancy complications, particularly benefiting densely populated developing countries with high PE incidence and limited access to routine prenatal care.

**Supplementary Information:**

The online version contains supplementary material available at 10.1186/s12884-025-07503-5.

## Background

Preeclampsia (PE) is a critical pregnancy complication marked by the emergence of hypertension after 20 weeks of gestation, often leading to multi-organ dysfunction in the mother. This condition is a significant global health concern, accounting for around 70,000 maternal deaths and 500,000 fetal and neonatal deaths each year [1–4]. Despite extensive research efforts utilizing maternal risk factors, mean arterial pressure, uterine artery pulsatility index, and various biochemical markers like pregnancy-associated plasma protein A, soluble vascular endothelial growth factor receptor 1, soluble endoglin, placental growth factor (PlGF), and soluble fms-like tyrosine kinase 1 (sFlt-1) [5–10], PE is frequently diagnosed late or missed, underscoring the necessity for more precise and early biomarkers and predictive tools.

Recently, circulating cell-free RNA (cfRNA) has emerged as a promising area of study. CfRNA consists of a diverse mixture of transcripts, including microRNA, long non-coding RNA, circular RNA, transfer RNA, and messenger RNA, derived from various cell types. Its association with numerous health conditions and presence in multiple body fluids have made cfRNA a valuable target for clinical applications such as bone marrow transplantation, neurodegeneration, cardiovascular diseases, oncology, and obstetrics [11–19]. A pivotal study by Quake et al. highlighted that a set of 18 cfRNA markers [20], identifiable between 5 to 16 weeks of gestation, could form the basis of a liquid biopsy test to predict potential PE cases well before symptoms appear. This correlation between cfRNA levels and organ health in PE suggests cfRNA’s potential as a vital biomarker.

Traditionally, cfRNA studies have focused on the overexpression and mutations of known genes, with polymerase chain reaction (PCR) being the primary technique used. However, the biomarker selection process for specific diseases remains largely inefficient and predominantly one-dimensional. Developing a comprehensive cfRNA data analysis approach could significantly enhance the use of extensive sequencing data, leading to more accurate early PE screening.

Artificial intelligence, particularly deep learning, offers promising advancements in medical predictions and diagnostics. These models, trained to learn from data, have been successfully applied in various medical fields such as interpreting chest radiographs, identifying hypertension, and classifying breast cancer [21–26]. Schmidt et al [27]. recently demonstrated that integrating extensive medical history, current condition, and laboratory data into machine learning algorithms, such as gradient-boosted trees and random forests, can effectively predict adverse PE outcomes. As more data is incorporated and algorithms are refined, the accuracy of these predictive models is expected to improve, making them invaluable tools for PE prediction.

**Fig. 1:**
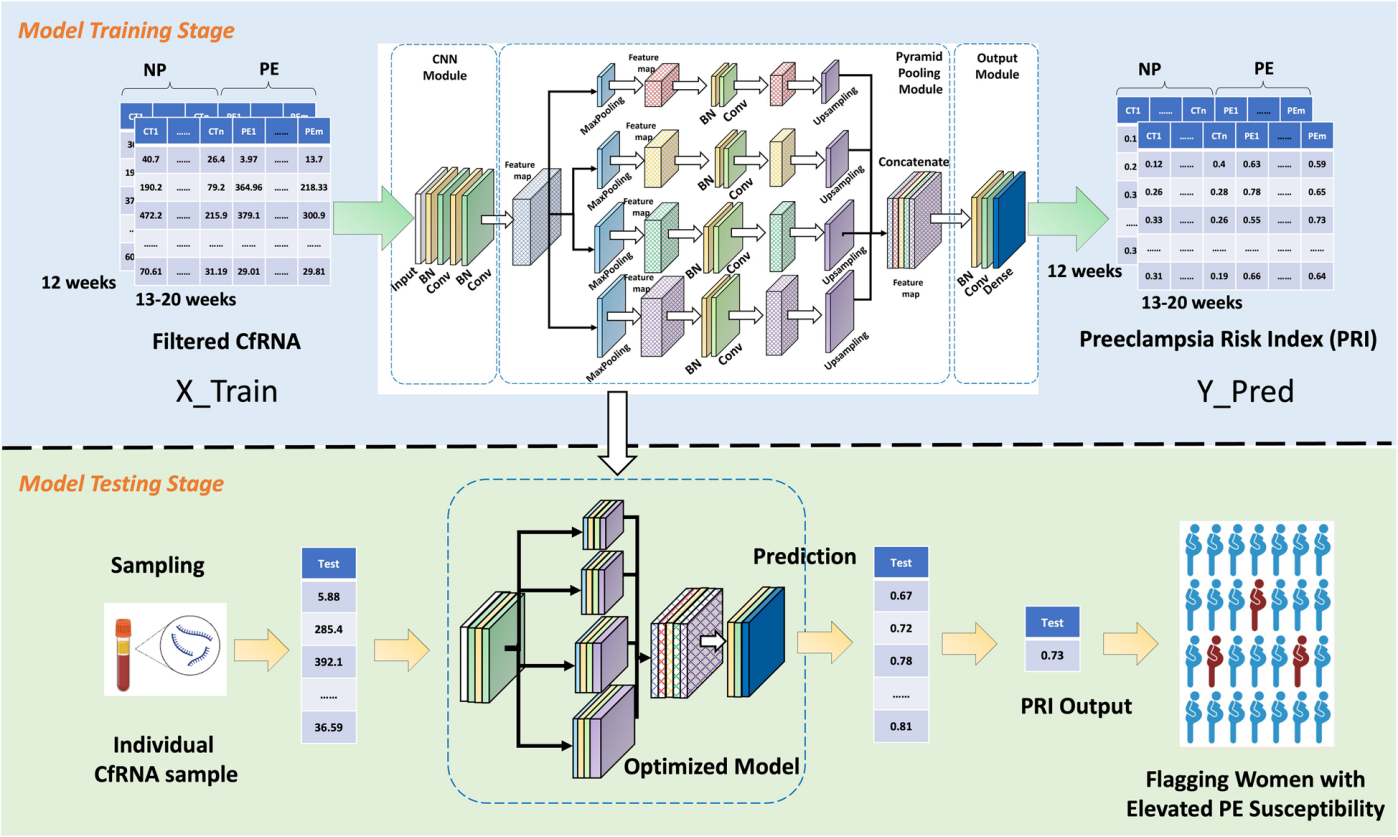
Cell-Free RNA and Pyramid Scene Parsing Network Based Early Preeclampsia Prediction. Maternal plasma cfRNAs undergo preprocessing to create datasets for both training and validation purposes. The trained network then predicts PE risk by analyzing variations in cfRNA profiles during early pregnancy. NP represents normal pregnancy, PE indicates preeclamptic pregnancy, and cfRNA stands for circulating cell-free RNA

In our research, we have developed a deep learning algorithm to calculate a Preeclamptic Risk Index (PRI) for pregnant women using cfRNA profiling. We implemented a Pyramid Scene Parsing Network (PSPNet) [28, 29], which achieved a remarkable alignment with the ground truth, exhibiting an average prediction error of 0.043 across 249 samples and a computational time of 10^–4^ s per sample. This innovative method facilitates rapid and precise PE risk assessment, offering significant potential to transform prenatal care by enabling timely intervention and personalized monitoring for at-risk pregnancies (Fig. 1).

## Methods

### Ethical statement

This study was exempt from ethics approval by the Ethical Committee of Xi’an Jiaotong University as it involved the analysis of publicly available data from the Gene Expression Omnibus (GSE192902) database and did not involve direct interaction with human subjects or animal models.

### Study design and prediction mechanism

**Fig. 2:**
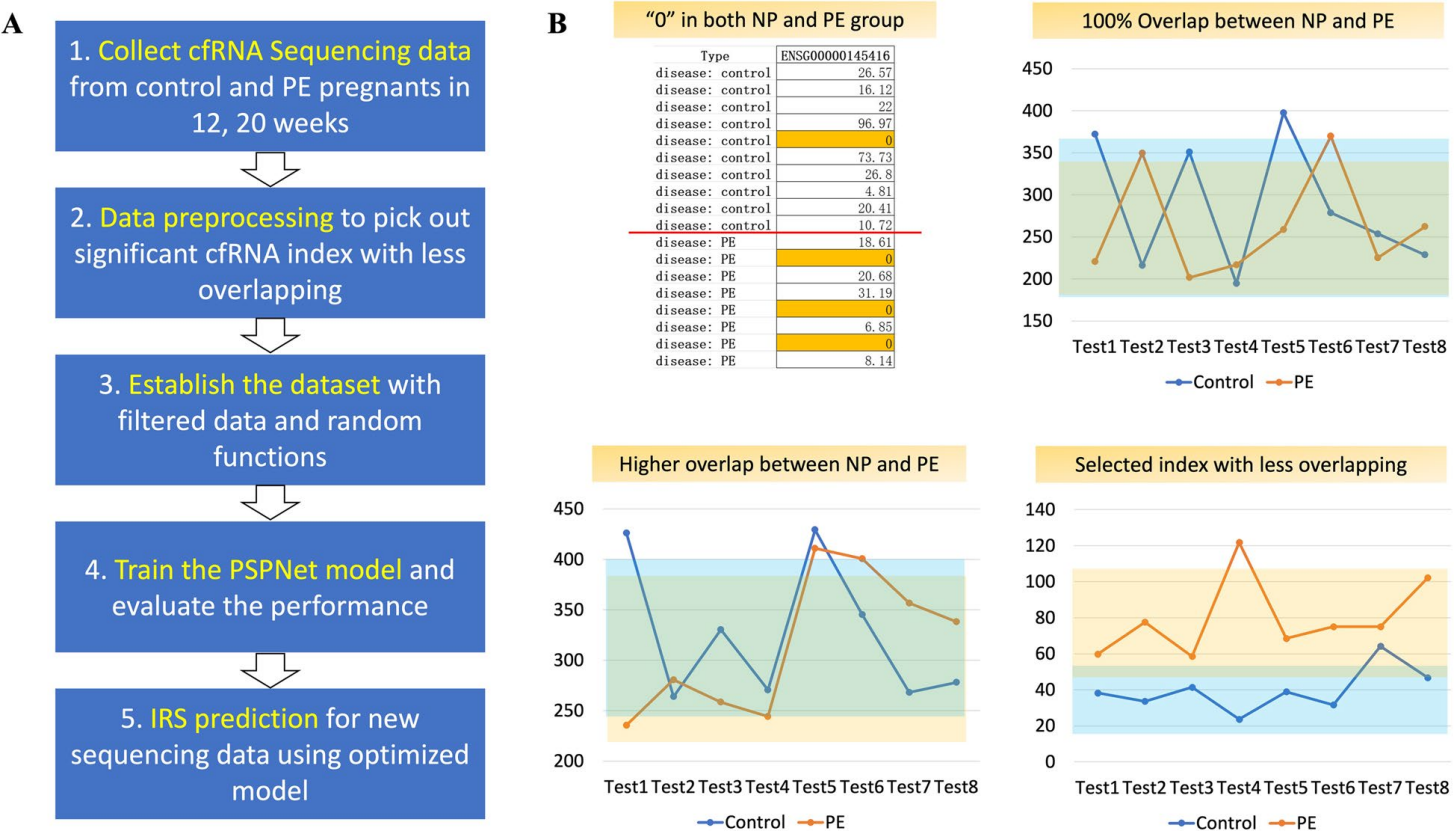
Overview of study design, data filtration, and model architecture. **A** Study design and prediction methodology. **B** Data filtration principles: (a) Zero expression in both PE and control groups; (b) Complete overlap in cfRNA expression domains; (c) Larger overlapping domains with smaller mean deviations; (d) Distributions with significant differences

To predict PE risk, we approached it as a data regression problem, creating a mapping between maternal plasma cfRNA profiles and probability vectors. Initially, we preprocessed the cfRNA sequencing data to filter out markers with significant differences between normotensive (NP) and preeclamptic (PE) groups. Based on the guidelines from the American College of Obstetricians and Gynecologists (ACOG), PE is defined as new-onset hypertension (systolic ≥ 140 mmHg or diastolic ≥ 90 mmHg) occurring after 20 weeks of gestation, accompanied by proteinuria or signs of end-organ dysfunction. Women with normotensive, uncomplicated pregnancies served as the normal control group. These filtered markers were used to construct training and validation datasets for our neural network model. The trained network predicts PE risk by generating a Preeclamptic Risk Index (PRI) based on variations in cfRNA profiles during early pregnancy (Fig. 2A).

### Real-World cfRNA profiling data

We obtained standardized and cleaned cfRNA sequencing data from the Gene Expression Omnibus (GSE192902) dataset. PE diagnosis was based on guidelines from the American College of Obstetrics and Gynecology (ACOG), and women with uncomplicated pregnancies served as the normal control group. Exclusion criteria ensured no participants had chronic hypertension or gestational diabetes. Additionally, the NP and PE groups were matched for race and ethnicity. Detailed demographic data was shown in Supplementary Table 1 and differences were analyzed using a chi-squared test for categorical variables and ANOVA for continuous variables. A total of 87 cfRNA profile sets were used as the training dataset, and 249 sets were used as the validation dataset.

### Data filtration

To establish a robust relationship between multidimensional cfRNA expression profiling and PE risk, we filtered the data to select cfRNAs with significant changes that could serve as PE risk indicators. The preprocessing of cfRNA sequencing data involved four key steps (Fig. 2B): The selection of significant cfRNA indicators is mainly based on the data mathematical statistics from the initial sequencing dataset. There are 4 rules for cfRNA selection in data preprocessing stage. 1) The cfRNA indicators both contain “0” expressive abundance in PE and NP group would not be suitable for PRI evaluation. It can be easily understood that PE probability contribution of this indicator will never be distinguished for PE or NP. 2) The indicator ranges of expressive abundance have 100% overlapping between PE and NP are not suitable for PRI evaluation. Because certain sequencing data may both contribute to the probability for the risks of PE and non-PE. 3) The indicators have overlapping rate below 0.6 between PE and NP distribution range are suitable for PE probability evaluation; 4) The mean values of indicator distribution ranges should have significant difference between PE and NP group. Here, the mean value difference is 1.5 ~ 2.0 multiple between PE and NP distribution ranges of this indicator. By leverage conditions (1), (2), (3), (4), those cfRNA indicators with significant difference in distribution ranges between PE and NP group are used for PRI evaluation. Each indicator contributes equal weight for the final PRI probability. Through analysis, data preprocessing is a tradeoff between the data dimensions in PRI evaluation and the filtered cfRNA indicators with significant differences. The more cfRNA indicators are selected, the more biological plausibility is taken into account, and the more scientific results will be obtained. On the other hand, more cfRNA indicators with lower differences are selected in the evaluation, the lower PRI discrimination will be produced between PE and NP probability. Therefore, the parameters/thresholds in data preprocessing stage are optimized by the final PRI result.

There are only 29 cfRNA indicators are selection from total 7163 sequencing data for 0 ~ 12 weeks pregnancy PRI evaluation and 25 cfRNA indicators for 13 ~ 20 weeks evaluation.

### Dataset generation and definition of Preeclamptic Risk Index (PRI)

To train our neural network model, we needed a substantial dataset. This was achieved by analyzing real-world cfRNA profiling data and generating a synthetic dataset [30–32]. The dataset construction process is detailed as follows:

Before filtering, cfRNAs in NP and PE groups were represented as CN [cfRNA, normal pregnancy] ([r, n]) and CP [cfRNA, preeclamptic pregnancy] ([r, p]), respectively. Initially, we had 7,160 cfRNAs, where “r” is the identification number for each cfRNA, and “n” and “p” are identifiers for participants in NP and PE groups.

After filtering, cfRNAs in NP and PE groups were represented as SN [selected cfRNA, normal pregnancy] ([s, n]) and SP [selected cfRNA, preeclamptic pregnancy] ([s, p]), respectively. Here, “s” denotes the number of cfRNAs post-filtration, determined by the parameters of the preprocessing algorithm.

Using these filtered cfRNAs, we built a training dataset for the proposed PSPNet model. The training dataset consisted of the parameters *x*_train_ and *y*_train_​, and the validation dataset comprised *x*_test_ and *y*_test_. In this context, *x*_train_ and *x*_test_ are the cfRNA expression matrices, and *y*_train_ and *y*_test_ are the corresponding probability vectors contributing to PE risk. The mean value of the PE probability vector was denoted as the PRI. Our dataset included two components: 1) actual cfRNA expression sequencing data from maternal peripheral blood and 2) values generated randomly using a Gaussian function. The expression quantities in *x*_train_ and *x*_test_ adhered to practical sequencing range distributions. The dataset generation using the Gaussian function is outlined in Eq. (1):

1 $$\begin{gathered} \left\{ {\begin{array}{*{20}c} {x\_train[i] = rands\left\{ {Max\left[ {Max\left( {SP[i]} \right),Max\left( {SN[i]} \right)} \right],Min\left[ {Min\left( {SP[i]} \right),Min\left( {SN[i]} \right)} \right],M} \right\}} \\ {x\_test[i] = rands\left\{ {Max\left[ {Max\left( {SP[i]} \right),Max\left( {SN[i]} \right)} \right],Min\left[ {Min\left( {SP[i]} \right),Min\left( {SN[i]} \right)} \right],Q} \right\}} \\ \end{array} } \right. \hfill \\ i \in [1,2,3, \cdots ,N] \hfill \\ \end{gathered}$$

In this equation, rands() denotes the Gaussian random function, Max() and Min() are the maximum and minimum value functions, and *M* and *Q* are the numbers of samples to be generated. We calculated the contribution of each cfRNA to the occurrence of PE or NP based on the expression levels and clinical diagnosis. For instance, if the expression of ENSG00000000460 was significantly higher in the PE group compared to the NP group, it was assigned a contribution value of “1” to PE.

The cfRNA contribution vector sets (*y*_train_ and *y*_test_) were computed from *x*_train_ and *x*_test_ and clinical diagnosis data. This process is described by Eqs. (2) and (3):

2 $$\begin{gathered} \left\{ {\begin{array}{*{20}c} {y\_train[i] = \frac{x\_train[i] - Min(x\_train[i])}{{Max(x\_train[i]) - Min(x\_train[i])}} \, , \, if \, avg(SP[i])> avg(SN[i])} \\ {y\_train[i] = \frac{Max(x\_train[i]) - x\_train[i]}{{Max(x\_train[i]) - Min(x\_train[i])}} \, , \, if \, avg(SP[i]) < avg(SN[i])} \\ \end{array} } \right. \hfill \\ i \in [1,2,3, \cdots N] \hfill \\ \end{gathered}$$

3 $$\begin{gathered} \left\{ {\begin{array}{*{20}c} {y\_test[i] = \frac{x\_test[i] - Min(x\_test[i])}{{Max(x\_test[i]) - Min(x\_test[i])}} \, , \, if \, avg(SP[i])> avg(SN[i])} \\ {y\_test[i] = \frac{Max(x\_test[i]) - x\_test[i]}{{Max(x\_test[i]) - Min(x\_test[i])}} \, , \, if \, avg(SP[i]) < avg(SN[i])} \\ \end{array} } \right. \hfill \\ i \in [1,2,3, \cdots N] \hfill \\ \end{gathered}$$

where avg() is the average value function, and *N* = *s*.

Ultimately, we obtained *x*_train_​ and *y*_train_ with dimensions [M, N] and *x*_test_ and *y*_test_ with dimensions [Q, N]. These values were reshaped to [1, N, M] and [1, N, Q], respectively, through dimension transformation. Here, *N* = *s*, *M* = 8000, and *Q* = 500, indicating that there were 8000 training sample vectors in 1 × N and 500 validation sample vectors in 1 × N. The PRI was computed as the average of *y*_test_. Table 1 provides details of the dataset.

**Table 1:** Dataset for PSPNet training and validation

Dataset	Dimension	Transformed Dimension	Note
x_train	[M, N] N = s	[1, N, M] M = 8000	Training dataset (cfRNA expression)
y_train	[M, N] N = s	[1, N, M] M = 8000	Training dataset (PE probability)
x_test	[Q, N] N = s	[1, N, Q] Q = 500	Validation dataset (cfRNA expression)
y_test	[Q, N] N = s	[1, N, Q] Q = 500	Validation dataset (PE probability)

### Pyramid Scene Parsing Network (PSPNet) construction

#### Model architecture

**Fig. 3:**
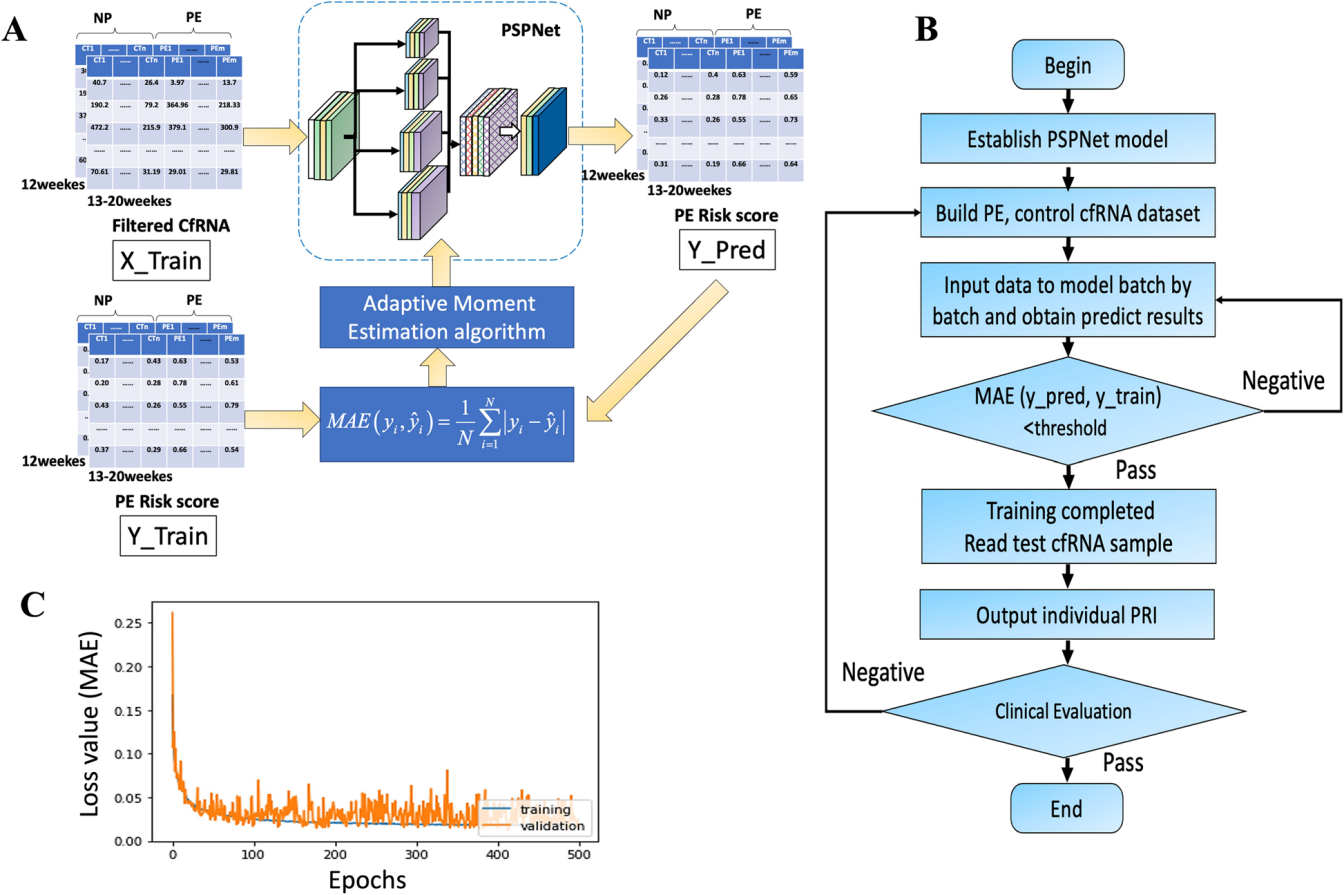
Pyramid Scene Parsing Network (PSPNet) Training and Construction. **A** The PSPNet architecture, consisting of an input layer, a series of residual convolution blocks, and output layers. Components include Conv (2D convolution), Dense (fully connected layer), BN (batch normalization), DP (dropout), MP (max pooling), ConvT (2D deconvolution), and TU (fine feature extraction modules). **B** Workflow for PE prediction using PSPNet. **C:** Loss value. X_Train: cfRNA expression matrices for the training dataset. Y_Train: Probability vectors corresponding to PE risk. Y_Pred: Predicted probability vectors generated by PSPNet. M × N: Dataset configuration with M profiles, each containing cfRNA indicators (filtered) of size 1 × N

The PSPNet was designed to predict PRI using cfRNA expressions from maternal peripheral blood samples collected within 12 weeks and between 13–20 weeks of gestation. The model comprises three primary modules: the Convolutional Neural Network (CNN) module, the Pyramid Pooling module, and the Output module (Fig. 3A, B).

#### CNN module

(1) The CNN module serves as the input layer, extracting feature maps from the cfRNA expression dataset and capturing low-level semantic information essential for subsequent layers; (2) Configuration: Convolutional kernel size is 3 × 3, with 128 channels and a stride of 5, Relu is adopted as activation function.

### Pyramid pooling module

(1) This module employs a pyramid structure to capture multi-scale global contextual features from each sub-region. Intermediate feature maps are further processed through max-pooling layers to produce refined feature maps at different scales. The convolutional layers extract semantic information from low to high levels; (2) Configuration: Convolutional kernel size is 3 × 3, with 128 channels and a stride of 5, Relu is adopted as activation function.

### Output module

(1) The Output module concatenates local pointwise features with learned multi-scale contextual features, resulting in more accurate predictions than using a baseline model alone. The final PE probability vector, generated by a dense layer, reveals the contributions of significant cfRNA expressions to PE risk; (2) Configuration: Convolutional kernel size is 3 × 3, with 128 channels and a stride of 5, Relu is adopted as activation function. The dense layer kernel size is 1 × 1, with 128 channels.

#### Preprocessing and data filtering

Before inputting the data into PSPNet, the cfRNA sequencing data undergo preprocessing to filter out cfRNAs with significant features. This step ensures that only the most relevant cfRNAs are used as input, with the predicted PE probability (PRI) as the output.

#### Overfitting prevention

To mitigate overfitting during model training, Batch Normalization layers are inserted before each convolutional layer to normalize input features. Additionally, a dropout operator with a parameter set to 0.25 is added after the convolutional layers.

#### Training configuration

The training of PSPNet is configured with the following hyperparameters (Fig. 3C). Optimizer: Adam; Learning Rate: 0.0005; Loss Function: Mean Absolute Error (MAE); Metrics: Accuracy; Batch Size: 16; Epochs: 500; Validation Split: 0.05.

The MAE loss function is defined as:

$$MAE\left( {y_{i} ,\hat{y}_{i} } \right) = \frac{1}{N}\sum\limits_{i = 1}^{N} {\left| {y_{i} - \hat{y}_{i} } \right|}$$

where *y*_*i*_ is the ground truth of PE probability, $\hat{y}_{i}$ is the predicted PE probability, and *N* is the number of samples.

Deep learning technique exerts advantages in data fitting, target classification, and information prediction. The more sample data used in training and validation stage, the better performance will be obtained in subsequent applications. Though sequencing data of NP and PE reaches 249 in total, the quantity is still limited for mainstream deep learning model to make optimal training and validation. Therefore, we take the following measures to enhance dataset and ensure the effectiveness. 1) Set multiple rules to select significant cfRNA indicators for prediction; 2) according to a distribution of those indicators, expand the sample data and dataset using Gaussian random function; 3) randomly select 30% real-world sequencing data and generate virtual dataset for model training and validation; 4) leverage 100% real-world sequencing data for final test and evaluation of proposed technique. The experimental dataset has non-overlap with those in training stage. All the results in this research article are based on the true sequencing data. Here, training-validation split (95–5) is just 95% samples in dataset for training and 5% for the validation of the loss value.

#### Computational efficiency

PSPNet provides an effective global contextual prior for single cfRNA expression-level scene parsing. The pyramid pooling module collects multi-level information more representatively than global pooling. PSPNet does not significantly increase computational cost compared to the original dilated Fully Convolutional Network (FCN). Both the global pyramid pooling module and the local FCN features are optimized simultaneously in end-to-end learning.

## Hardware configuration

The PSPNet model was trained and validated on a computer with the following specifications. CPU: Intel Core i5 13600 K 3.5 GHz 14C/20 T; RAM: DDR4 3000 MHz 32 GB; GPU: Nvidia RTX2080Ti 11 GB; Storage: SSD M.2 3600 Mb/s 1 TB. This hardware configuration ensured efficient handling of the computational demands during the PSPNet training process.

## Results

### Data filtration for cfRNA indicators

A total of 7,160 cfRNAs were initially detected and subsequently filtered through multiple tests and parameter optimizations, we identified cfRNAs with significant differences between the NP and PE groups. Specifically, 29 cfRNAs were selected as PE indicators for samples collected at ≤ 12 weeks of gestation (gws), and 25 cfRNAs were chosen for samples collected at 13–20 gws (Table 2).

**Table 2:** Filtered cfRNA indicators for different sampling time

Sampling time	Gene	ENSEMBL	Name	Biological process[GO]	Molecular function[GO]
**cfRNA indicators before or at 12 gestational weeks**		ENSG00000213846			
	GSPT1	ENSG00000103342	G1 to S phase transition 1	translational elongation	GTPase activity,translation factor activity,RNA binding
		ENSG00000265764			
	RN7SL573P	ENSG00000239607	RNA, 7SL, cytoplasmic 573, pseudogene		
	RN7SL752P	ENSG00000239437	RNA, 7SL, cytoplasmic 752, pseudogene	SRP-dependent cotranslational protein targeting to membrane, signal sequence recognition	
	APOL6	ENSG00000221963	Apolipoprotein L6	involved in lipid transport	lipid binding
	IGF2	ENSG00000167244	insulin like growth factor 2	regulation of gene expression,gene expression,nervous system development	mRNA 3’-UTR binding
	RN7SL151P	ENSG00000244230	RNA, 7SL, cytoplasmic 151, pseudogene		
	RN7SL5P	ENSG00000265735	RNA, 7SL, cytoplasmic 5, pseudogene		
	RN7SL767P	ENSG00000241529	RNA, 7SL, cytoplasmic 767, pseudogene	SRP-dependent cotranslational protein targeting to membrane,signal sequence recognition	
	BNIP3L	ENSG00000104765	BCL2 interacting protein 3 like	regulation of programmed cell death,mitochondrial transport,regulation of membrane permeability,apoptotic signaling pathway,defense response to virus,apoptotic mitochondrial changes,mitochondrial membrane organization	protein binding,lamin binding,identical protein binding,protein homodimerization activity
	IRS2	ENSG00000185950	insulin receptor substrate 2	insulin receptor signaling pathway	signaling receptor binding,protein-containing complex binding,phosphatidylinositol 3-kinase binding
	RN7SL381P	ENSG00000263968	RNA, 7SL, cytoplasmic 381, pseudogene		
	RN7SL665P	ENSG00000264169	RNA, 7SL, cytoplasmic 665, pseudogene		
	RNA5-8SP2	ENSG00000200434	RNA, 5.8S ribosomal pseudogene 2		structural constituent of ribosome
	CTSB	ENSG00000164733	cathepsin B	proteolysis involved in cellular protein catabolic process	cysteine-type endopeptidase activity
	MT-TR	ENSG00000210174	mitochondrially encoded tRNA arginine		
	RN7SL396P	ENSG00000244642	RNA, 7SL, cytoplasmic 396, pseudogene		
	RN7SL674P	ENSG00000239899	RNA, 7SL, cytoplasmic 674, pseudogene	SRP-dependent cotranslational protein targeting to membrane, signal sequence recognition	
	RNA5-8SP6	ENSG00000251705	RNA, 5.8S ribosomal pseudogene 6		structural constituent of ribosome
	FBXO7	ENSG00000100225	F-box protein 7	autophagy of mitochondrion,ubiquitin-dependent protein catabolic process,protein targeting to mitochondrion,protein targeting to mitochondrion,protein targeting to mitochondrion	ubiquitin-protein transferase activity,protein binding,protein kinase binding,ubiquitin protein ligase binding,ubiquitin binding
	NFKBIA	ENSG00000100906	NFKB inhibitor alpha	protein import into nucleus,apoptotic process,Notch signaling pathway,I-kappaB kinase/NF-kappaB signaling,I-kappaB kinase/NF-kappaB signaling	protein binding,protein binding,enzyme binding,ubiquitin protein ligase binding,identical protein binding
	RN7SL4P	ENSG00000263740	RNA, 7SL, cytoplasmic 4, pseudogene	SRP-dependent cotranslational protein targeting to membrane, signal sequence recognition	
	RN7SL736P	ENSG00000275803	RNA, 7SL, cytoplasmic 736, pseudogene		
	RNA5SP202	ENSG00000201185	RNA, 5S ribosomal pseudogene 202		structural constituent of ribosome
	RNA5SP267	ENSG00000201763	RNA, 5S ribosomal pseudogene 267		structural constituent of ribosome
	TMEM185A	ENSG00000269556	transmembrane protein 185A		protein binding
	WARS1	ENSG00000140105	tryptophanyl-tRNA synthetase 1	cellular amino acid metabolic process,tRNA metabolic process,translational elongation	ligase activity,catalytic activity,acting on RNA
	Y RNA	ENSG00000206659	Y RNA		
**cfRNA indicators between 13 to 20 gestational weeks**	BCL6	ENSG00000113916	BCL6 transcription repressor	negative regulation of	Protein binding,protein binding,DNA-binding transcription factor activity
	CSF3R	ENSG00000119535	colony stimulating factor 3 receptor	cell adhesion, cytokine-mediated signaling pathway,regulation of myeloid cell differentiation, neutrophil chemotaxis, signal transduction	cytokine binding, granulocyte colony-stimulating factor binding, cytokine receptor activity
	FCN1	ENSG00000085265	ficolin 1	cell surface pattern recognition receptor signaling pathway, complement activation, lectin pathway, G protein-coupled receptor signaling pathway,	protein binding, G protein-coupled receptor binding, carbohydrate derivative binding, sialic acid binding
	MALAT1	ENSG00000251562	metastasis associated lung adenocarcinoma transcript 1	positive regulation of cell motility	
	N4BP1	ENSG00000102921	NEDD4 binding protein 1	negative regulation of proteasomal ubiquitin-dependent protein catabolic process, RNA phosphodiester bond hydrolysis, cellular response to UV	protein binding, mRNA binding, ribonuclease activity, ubiquitin binding
	NEAT1	ENSG00000245532	Nuclear paraspeckle assembly transcript 1	miRNA-mediated gene silencing	protein binding
	NFAM1	ENSG00000235568	NFAT activating protein with ITAM motif 1	B cell differentiation, positive regulation of DNA-binding transcription factor activity, intracellular signal transduction	protein binding, transmembrane signaling receptor activity
	PTPRC	ENSG00000081237	Protein tyrosine phosphatase receptor type C	T cell receptor signaling pathway, negative regulation of protein kinase activity, regulation of receptor signaling pathway via JAK-STAT, B cell differentiation	protein binding, protein tyrosine phosphatase activity, heparan sulfate proteoglycan binding
	RN7SL128P	ENSG00000240869	RNA, 7SL, Cytoplasmic 128, Pseudogene		
	RN7SL230P	ENSG00000264916	RNA, 7SL, cytoplasmic 230, pseudogene		
	RN7SL5P	ENSG00000265735	RNA, 7SL, Cytoplasmic 5, Pseudogene		
	RN7SL665P	ENSG00000264169	RNA, 7SL, cytoplasmic 665, pseudogene		
	RN7SL674P	ENSG00000239899	RNA, 7SL, Cytoplasmic 674, Pseudogene		
	RN7SL736P	ENSG00000275803	RNA, 7SL, cytoplasmic 736, pseudogene		
	RN7SL752P	ENSG00000239437	RNA, 7SL, cytoplasmic 752, pseudogene		
	RNA5SP202	ENSG00000201185	RNA, 5S ribosomal pseudogene 202		
	RNA5SP242	ENSG00000201041	RNA, 5S ribosomal pseudogene 242		
	RNA5SP267	ENSG00000201763	RNA, 5S ribosomal pseudogene 267		
	RNA5SP290	ENSG00000252942	RNA, 5S ribosomal pseudogene 290		
	RNA5SP355	ENSG00000202187	RNA, 5S Ribosomal Pseudogene 355		
	RNA5SP481	ENSG00000252623	RNA, 5S ribosomal pseudogene 481		
	RNA5SP74	ENSG00000200408	RNA, 5S ribosomal pseudogene 74		
	RPGR	ENSG00000156313	retinitis pigmentosa GTPase regulator	intraciliary transport, visual perception, intracellular protein transport, regulation of catalytic activity, response to stimulus	protein binding, RNA binding, guanyl-nucleotide exchange factor activity
	SMCHD1	ENSG00000101596	structural maintenance of chromosomes flexible hinge domain containing 1	nose development, chromosome organization, double-strand break repair, inactivation of X chromosome by heterochromatin formation	protein binding, ATP binding, ATP hydrolysis activity, protein homodimerization activity

### Calculation of PRI ground truth

Clinical diagnosis outcomes for enrolled women were classified as either NP or PE. The PRI for women diagnosed with PE was defined as “1,” while for those with NP, it was defined as “0”. The PRI for each participant was calculated accordingly. Using Eqs. (2) and (3) to process the cfRNA profiles, we derived the PRI for each sampling time, which served as the “calculated PRI” or ground truth.

**Fig. 4:**
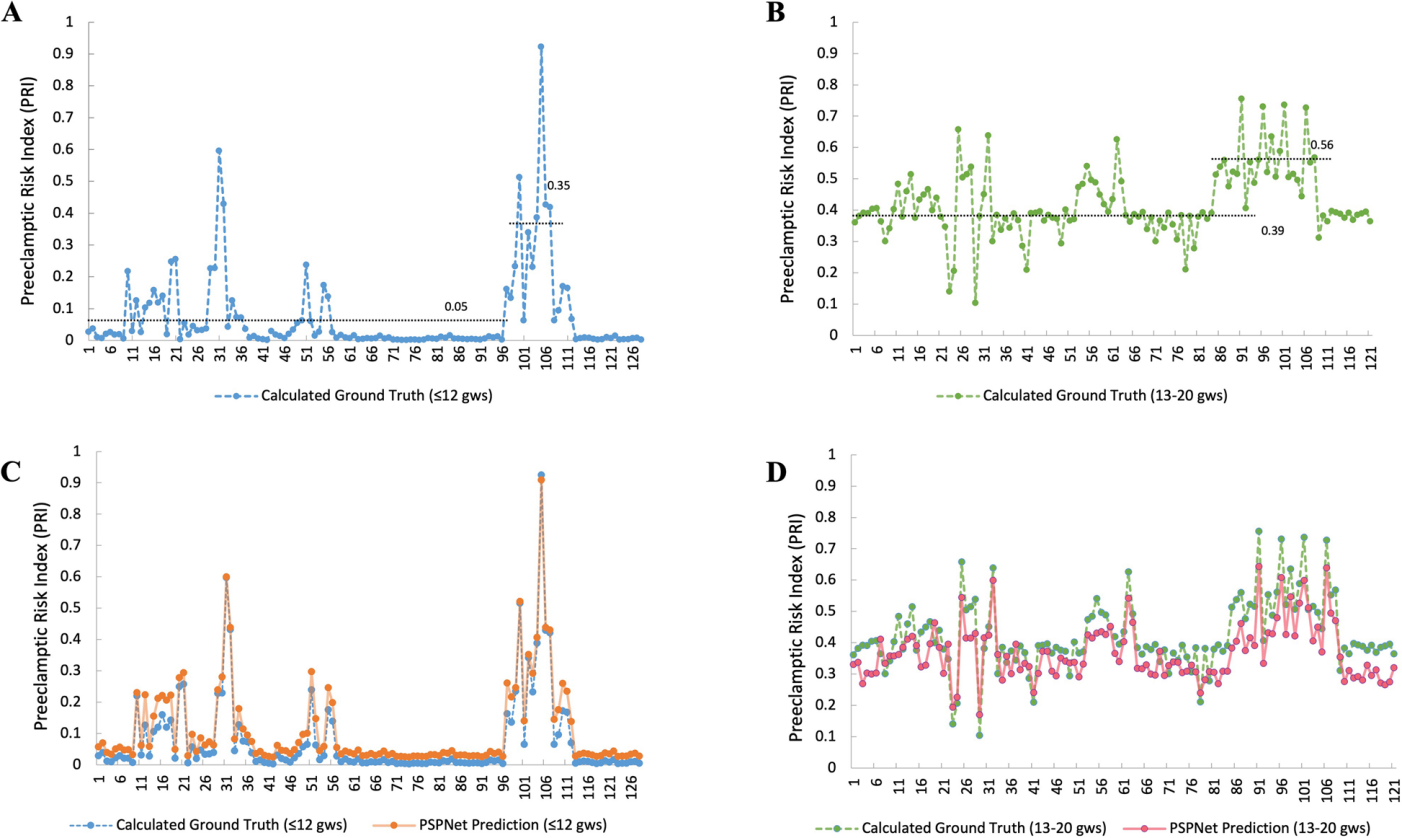
The results of PRI by PSPnet. **A** Ground truth PRI for cfRNA profiles sampled at ≤ 12 gws. **B** Ground truth PRI for cfRNA profiles sampled between 13 and 20 gws. **C** PSPNet-predicted PRI for cfRNA profiles sampled at ≤ 12 gws. **D** PSPNet-predicted PRI for cfRNA profiles sampled between 13 and 20 gws. PSPNet, Pyramid Scene Parsing Network; gws, gestational weeks; PRI, Preeclamptic Risk Index

For samples collected at ≤ 12 gws, the average calculated PRI was 0.05 for the NP group and 0.35 for the PE group (Fig. 4A). For samples collected at 13–20 gws, the average calculated PRI was 0.39 for the NP group and 0.56 for the PE group (Fig. 4B). The distinct differences in the average calculated PRI between NP and PE groups underscore the effectiveness of the filtered cfRNA indicators in distinguishing between these two conditions, supporting the model’s clinical applicability.

### PSPNet-based PRI verification

Training and validation of the PSPNet model reduced MAE of probability prediction to 0.019, resulting in an optimized model. To validate the model’s accuracy, real-world cfRNA expression data were used as the input set *x*_test​_ for the PSPNet model to obtain the PSPNet-based PRI. This PRI was then compared with the ground truth (calculated PRI) from real-world cfRNA profiling. For samples collected at ≤ 12 gws, the predicted PSPNet-based PRI closely matched the ground truth, indicating the model’s fitting ability (Fig. 4C). Abscissa axis of figure is the sample index under prediction and the value of each point is the corresponding PE PRI. The MAE between the prediction and ground truth was only 0.0178, effectively distinguishing PE from NP using the average PSPNet-based PRI. Similarly, for samples collected at 13–20 gws, the PSPNet predictions approximated the ground truth, with an MAE of only 0.0195 (Fig. 4D).

### Prediction error and time efficiency of PSPNet

**Fig. 5:**
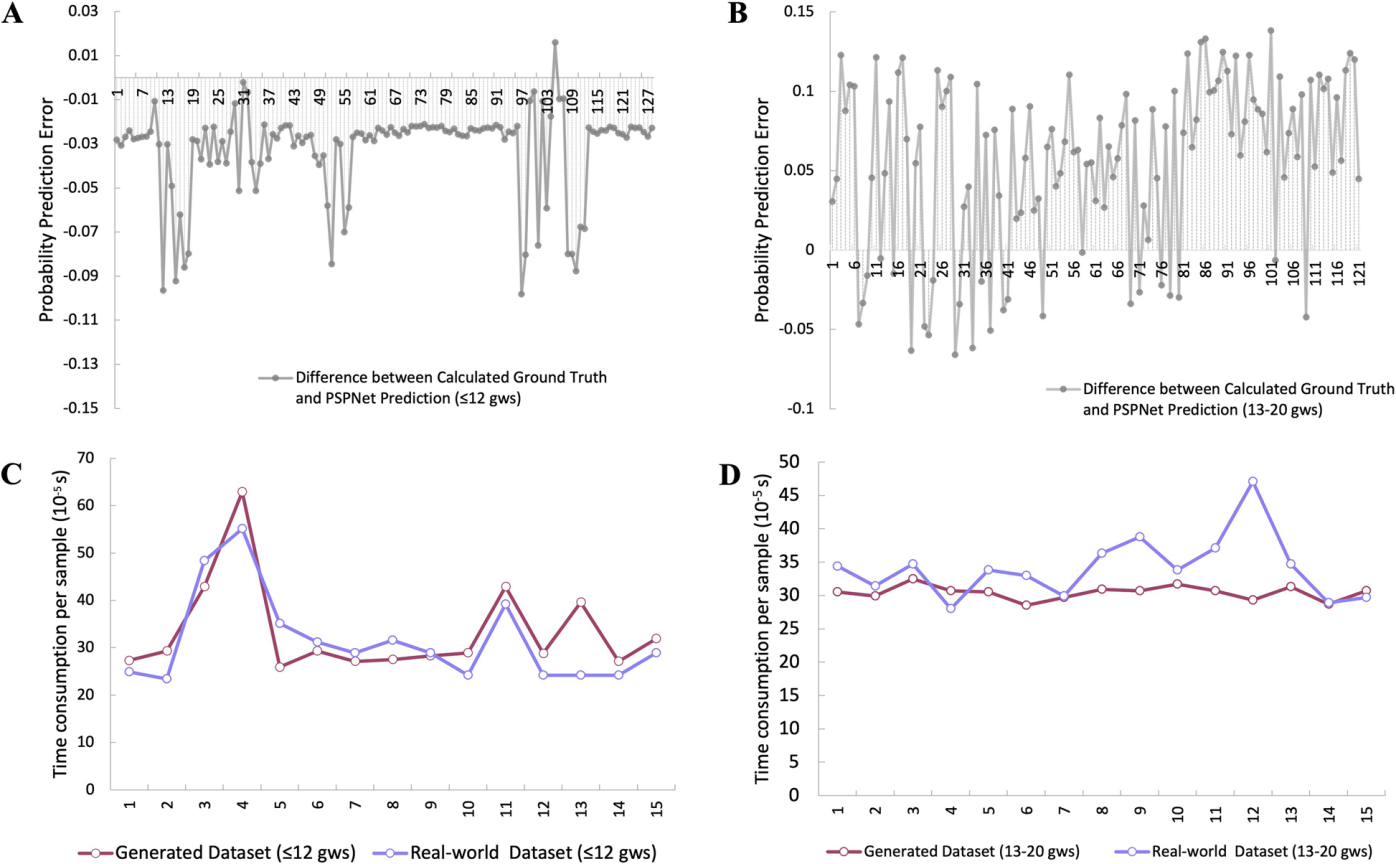
The Parameters of PSPNet. **A** Error amplitude of PSPNet-predicted PRI for cfRNA profiles sampled at ≤ 12 gws. **B** Error amplitude of PSPNet-predicted PRI for cfRNA profiles sampled between 13 and 20 gws. **C** Processing efficiency for cfRNA profiles sampled at ≤ 12 gws. **D** Processing efficiency for cfRNA profiles sampled between 13 and 20 gws. PRI stands for Preeclamptic Risk Index; gws stands for gestational weeks

The error amplitude of the PSPNet-based PRI for cfRNA samples collected at ≤ 12 gws is shown in Fig. 5A. The maximum absolute error, peak-to-valley (PV) error, and mean absolute error were 0.098, 0.114, and 0.032, respectively. For samples collected at 13–20 gws (Fig. 5B), the maximum absolute error was 0.13, the PV error was 0.195, and the mean absolute error was 0.055. Overall, the prediction error for PRI was well-contained within a small range, demonstrating the PSPNet model’s strong data-fitting capabilities.

We also evaluated the processing efficiency for large datasets from population screenings, using the prediction time efficiency as a benchmark. The time required to output a PRI value was recorded for cfRNA profiling samples fed into the trained PSPNet model. As shown in Fig. 5C and D, across 15 consecutive experiments, the average time to output a PRI was 10^–4^ s per sample.

### Comparative experiments and comprehensive evaluation

**Fig. 6:**
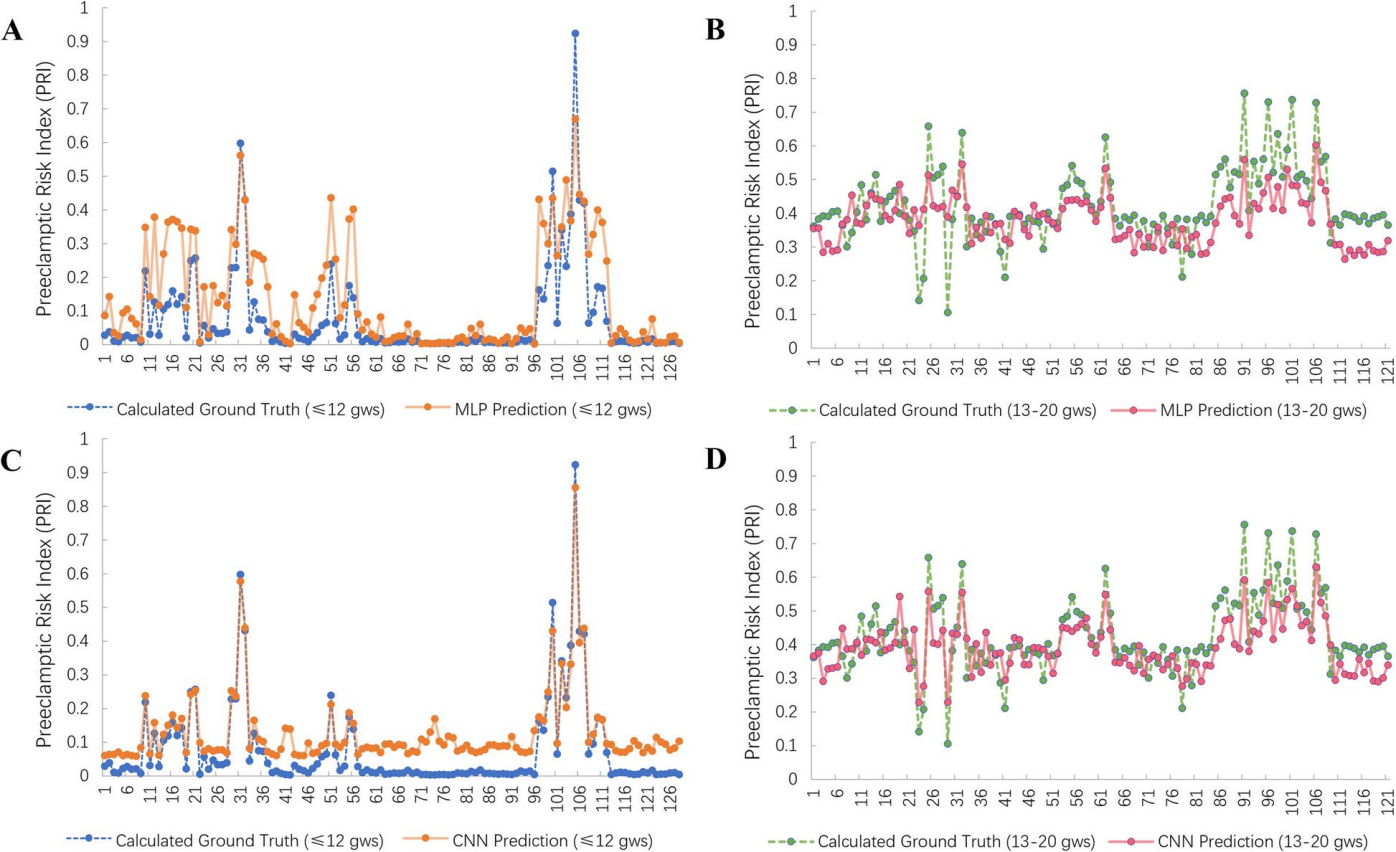
The results of PRI by MLP and CNN. **A** Ground truth PRI for cfRNA profiles sampled at ≤ 12 gws by MLP. **B** Ground truth PRI for cfRNA profiles sampled between 13 and 20 gws by MLP. **C** PSPNet-predicted PRI for cfRNA profiles sampled at ≤ 12 gws by CNN. **D** PSPNet-predicted PRI for cfRNA profiles sampled between 13 and 20 gws by CNN. gws, gestational weeks; PRI, Preeclamptic Risk Index

To further evaluate the effectiveness of proposed method, we have added comparative experiments to test the prediction performance in multi-dimensions. Convolutional neural network (CNN) and Multilayer Perceptron (MLP) are wildly applied techniques in data prediction, classification and fitting, that play key roles in sequencing data analysis. The same way, 12 gws and 13–20 gws dataset are engaged in the prediction tests by leveraging CNN and MLP whose corresponding results can be compared with that of proposed method (Fig. 4C and D). Figure 6A and B show the prediction results of 12 gws and 13–20 gws groups that produced by MPL; Fig. 6C and D shows the prediction results of 12 gws and 13–20 gws groups that produced by CNN. Abscissa axis of each plot is the sample index under prediction and the value of each point is the corresponding PE PRI. It can be found from the plot that predicted PRI still have significant difference with the ground truth in both 12gws and 13-20gws. While CNN produces a more similar trend than MLP in PRI distributions. MLP predicts more accurate PRI in NP samples and CNN model have advantages in PE PRI prediction.

From Fig. 4C and D, it can be found that the proposed method shows the smallest error between prediction results and ground truth in both 12 gws and 13 ~ 20 gws data. It shows excellent performance in both NP and PE PRI predictions. While in prediction accuracy, CNN has a better performance than MLP, especially for higher PRI samples. Based on the comparative experiments, proposed model, Convolutional neural network (CNN) and Multilayer Perceptron (MLP) are all join the comprehensive evaluation under the matric of MAE, Precision, Recall, AUC, ROC curve, and F1-score. Here, comprehensive evaluation results on 12 gws and 13–20 gws data are shown in the Supplementary Table 3 and Supplementary Table 4 respectively. All the superior data in the tables are enhanced in each method and matric. The less MAE score is obtained the smaller prediction error will be produced. As for the Precision, Recall, AUC, ROC curve, and F1-score, the higher scores are gained the more accurate classifier will be attained.

**Fig. 7:**
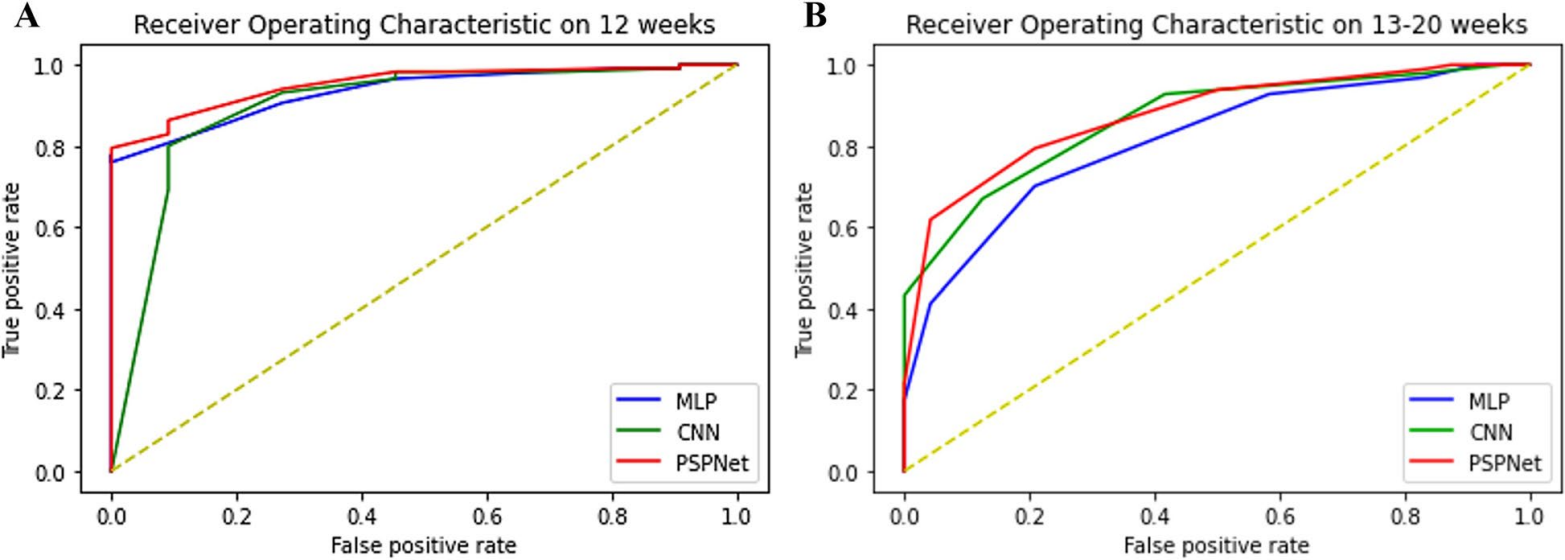
Receiver operating characteristics of prediction models. **A** Receiver operating characteristics ≤ 12 gws; **B** Receiver operating characteristics between 13 and 20 gws; gws, gestational weeks

Concluded from the comparative experiments, the proposed show the excellent performance in PRI prediction. In the evaluation of classifier effectiveness, Receiver Operating Characteristic are shown in the Fig. 7 reflects the classification performance of each model. Overall, the proposed method has a higher ROC curve and larger AUC in diagrams than others, which shows better classification effective in PE samples.

## Discussion

In this study, we identified 29 cfRNAs as indicators of PE for samples sequenced at ≤ 12 gws and 25 cfRNAs for samples sequenced at 13–20 gws. During the training and validation phases, we developed a PSPNet model that processes cfRNA profiling data to generate the PRI. The MAE between the predicted PRI and the ground truth was 0.0178 for cfRNA data sampled at ≤ 12 gws and 0.0195 for data sampled at 13–20 gws. The predicted PRI values closely matched the ground truth, with maximum absolute error values of 0.098 and 0.13, PV error values of 0.114 and 0.195, and mean absolute errors of 0.032 and 0.055 for samples at ≤ 12 gws and 13–20 gws, respectively. Additionally, the average prediction time for PRI was 10^–4^ s per sample. These results demonstrate the strong fitting ability of our PSPNet model, suggesting its potential for effective clinical implementation to predict PE before 20 gws almost instantaneously for individual patients.

Early prediction of PE significantly enhances prophylactic measures, benefiting maternal and neonatal healthcare. Quake et al. constructed a logistic regression model with an elastic net penalty and identified a panel of 18 cfRNAs from 5 to 16 gws, forming the basis of a liquid biopsy test for early PE detection [31]. Compared to the sFlt-1/PlGF ratio used in mid-gestation PE prediction [32], cfRNA offers earlier and more sensitive predictive capabilities. Our approach involved downloading cfRNA profiles and applying our data filtration principles, resulting in the selection of 29 cfRNAs as PE risk indicators for samples sequenced at ≤ 12 gws and 25 cfRNAs for samples sequenced at 13–20 gws. Unlike previous studies, we did not filter the same cfRNAs identified by Quake et al. Our analysis revealed common cfRNAs (RN7SL5P, RN7SL665P, RN7SL674P, RN7SL736P, RN7SL752P, RNA5SP202, and RNA5SP267) across both time points, suggesting further investigation into their roles in PE pathogenesis.

Advancements in artificial intelligence have also contributed to PE detection and diagnosis. Maric et al. developed a machine learning-based PE prediction model using statistical learning methods to analyze clinical and laboratory data from routine prenatal visits, achieving an area under the curve (AUC) of 0.89 for early-onset PE prediction. Schmidt et al. integrated real-world medical history, current condition, and laboratory variables into a machine learning-based algorithm using gradient-boosted tree and random forest models [27]. These examples underscore the potential of machine learning to integrate conventional maternal risk factors, biophysical markers, and maternal plasma protein levels in PE prediction. In contrast, our PSPNet model, a deep learning algorithm distinct from statistical learning methods, demonstrated significant advantages in multi-object classification, image segmentation, data fitting, and prediction. Unlike previous studies relying on medical history and laboratory variables as input data, we utilized novel cfRNA biomarkers to train and evaluate our PSPNet model.

Our study achieved an average prediction time of 10^–4^ s per sample, a metric not addressed in previous research. This rapid prediction capability is crucial for processing large datasets from population screenings, allowing thousands of sequencing data points to be analyzed in seconds. This high-speed prediction method is suitable for clinical practice as an auxiliary diagnostic tool, particularly in remote rural areas with limited access to prenatal care.

The integration of sensitive cfRNA biomarkers with our PSPNet model facilitates consistent evaluation of PRI from the first clinical prenatal visit. This approach enables continuous monitoring of PE risk and serves as a comprehensive response indicator for prophylactic treatments in pregnant women. Given the PSPNet model’s rapid processing time of 10^–4^ s per sample, it is feasible to implement a cloud laboratory system to predict PE from cfRNA profiling samples across China, providing early warnings for women with hidden-onset PE, especially in remote areas.

Our findings suggest several avenues for future research. Integrating medical history, laboratory variables, and additional biomarkers into the current PSPNet model could enhance the accuracy of PRI predictions for pregnant women, enabling precise PE risk assessment at any gestational week. As cfRNA profiling is associated with PE-related tissue and organ function, the model could be modified to provide warnings about specific tissues or organs compromised by PE development. With the rapid advancement of AI and the increasing use of public databases in healthcare research, it is crucial to ensure patient privacy protection and responsible data usage. Additionally, the future of medical artificial intelligence will require improved clinical data availability and interoperability, necessitating the construction of large-scale medical information and data storage facilities.

This study introduces a novel deep learning-based PSPNet model using sensitive cfRNA biomarkers, providing a foundation for further research. The model can evaluate the PRI of pregnant women as early as 12 gws, earlier than previous methods. The prediction error of our PSPNet model is well-controlled within 0.043 (mean value), and the processing time is only 10^–4^ s, indicating excellent potential for clinical application.

However, the study has limitations. Deep learning models are prone to fitting errors and may overfit the training data due to their high dimensionality. Additionally, the small size of real-world cfRNA profiling data poses challenges, as no algorithm can fully replicate human-derived sequencing profiles. Factors such as hereditary differences, individual variations, preanalytical conditions, background noise, quantification strategies, batch effects, and operational errors can affect cfRNA levels, compromising reproducibility, interpretability, and specificity.

## Conclusions

In this study, we utilized novel cfRNA biomarkers in conjunction with a PSPNet model to develop a reliable PRI for predicting PE. This approach demonstrates significant potential for rapid, minimally invasive monitoring of individual PE risk. The integration of cfRNA biomarkers and advanced deep learning techniques facilitates early detection and continuous risk assessment, contributing to enhanced maternal and neonatal healthcare. The PSPNet model’s high accuracy, low prediction error, and rapid processing time position it as a valuable tool for clinical applications, especially in regions with limited access to prenatal care.

## Supplementary Information

Supplementary Material 1.

Supplementary Material 2.

Supplementary Material 3.

## Data Availability

The data underlying this article are available in the article and in its online supplementary material. The cfRNA employed in current study were downloaded from the Gene Expression Omnibus (GSE192902).

## Abbreviations

cfRNA: Circulating cell-free RNA
PE: Preeclampsia
PSPNet: Pyramid Scene Parsing Network
PRI: Preeclamptic Risk Index
NP: Control group
MAE: Mean absolute error
