# Correction: Exploring the potential of cell‑free RNA and pyramid scene parsing network for early preeclampsia screening

**DOI:** 10.1186/s12884-025-08011-2

**Published:** 2025-08-12

**Authors:** Zhuo Zhao, Xiaoxu Liu, Yonghui Guan, Chunfang Li, Zheng Wang

**Affiliations:** 1 Key Laboratory of Shaanxi Province for Craniofacial Precision Medicine Research, College of Stomatology, Xi’an Jiaotong University, No.98, Xiwu Road, Xi’an, Shaanxi People’s Republic of China; 2 Clinical Research Center of Shaanxi Province for Dental and Maxillofacial Diseases, College of Stomatology, Xi’an Jiaotong University, No.98, Xiwu Road, Xi’an, Shaanxi People’s Republic of China; 3 State Key Laboratory for Manufacturing System Engineering, Xi’an Jiaotong University, Xi’an, China; 4 Department of Physiology and Pathophysiology, School of Basic Medical Sciences, Xian Jiaotong University, Xi’an, China; 5 Department of Urology, The First Affiliated Hospital of Xinjiang Medical University, Urumqi, China; 6 Department of Obstetrics & Gynecology, The First Affiliated Hospital of Xi’an Jiaotong University, No.76, Yanta West Road, Xi’an, Shaanxi People’s Republic of China

**Correction: BMC Pregnancy Childbirth 25**,** 445 (2025)**

**https://doi.org/10.1186/s12884-025-07503-5**

Following publication of the original article [1], the authors would like to correct the number of participants in the second paragraph under the heading **Dataset generation and definition of Preeclamptic Risk Index (PRI)**.

The sentence currently reads:

neural network model, we needed a substantial dataset. This was achieved by analyzing realworld cfRNA profiling data and generating a synthetic dataset [30–32]单击或点击此处输入文字。.

The sentence should read:

neural network model, we needed a substantial dataset. This was achieved by analyzing realworld cfRNA profiling data and generating a synthetic dataset [30–32].

The original article [1] has been corrected.
