# Supplementary material for: Exploring the potential of cell-free RNA and Pyramid Scene Parsing Network for early preeclampsia screening
Source: BMC Pregnancy Childbirth. 2025 Apr 14;25:445. doi: 10.1186/s12884-025-07503-5 (PMC11995606; doi:10.1186/s12884-025-07503-5)
Supplement: Supplementary file 1 — Supplementary Material 1. [file 12884_2025_7503_MOESM1_ESM.docx]

| **Characteristic** | **NP (Normotensive)** | **PE (Preeclampsia)** |
| --- | --- | --- |
| Sample size (n) | 180 | 69 |
| Maternal age (years, Mean ± SD) | 31 ± 5 | 32 ± 6 |
| Nulliparous (% (n)) | 28% (50) | 34% (24) |
| Smoker (% (n)) | 3% (5) | 0% (0) |
| History of PTB (% (n)) | 10% (17) | 26% (18) |
| History of PE (% (n)) | 3% (5) | 19% (13) |
| Ethnicity (% (n)) |  |  |
| - White | 69% (95) | 57% (33) |
| - Asian | 16% (22) | 15% (10) |
| - Hispanic | 20% (28) | 24% (17) |
| - Black | 5% (7) | 4% (3) |
| - Other | 2% (3) | 1% (1) |
| - Unknown | 1% (2) | 8% (5) |
| Gestational age at delivery (weeks, Mean ± SD) | 39 ± 1 | 36 ± 2 |
| Mode of delivery (vaginal, % (n)) | 65% (116) | 50% (35) |
| Preterm birth (PTB, % (n)) | 0% (0) | 38% (26) |
| Fetal sex (male, % (n)) | 54% (97) | 56% (38) |
| Fetal weight (kg, Mean ± SD) | 3.4 ± 0.4 | 3.0 ± 0.6 |
| Small for gestational age (SGA, % (n)) | 0% (0) | 5% (3) |

**Supplementary Table 1. Participant Demographics and Clinical Characteristics**
