# Supplementary material for: Exploring the potential of cell-free RNA and Pyramid Scene Parsing Network for early preeclampsia screening
Source: BMC Pregnancy Childbirth. 2025 Apr 14;25:445. doi: 10.1186/s12884-025-07503-5 (PMC11995606; doi:10.1186/s12884-025-07503-5)
Supplement: Supplementary file 2 — Supplementary Material 2. [file 12884_2025_7503_MOESM2_ESM.docx]

**Supplementary Table 2 Evaluation results on 12 gws data in multiple metric**

| **Method** | **MAE** | **Precision** | **Recall** | **AUC** | **F1 Score** |
| --- | --- | --- | --- | --- | --- |
| PSPNet | **0.032** | **0.958** | 0.983 | **0.9386** | **0.970** |
| MLP | 0.071 | 0.927 | **0.991** | 0.9191 | 0.958 |
| CNN | 0.056 | 0.942 | 0.980 | 0.8624 | 0.961 |
