# Supplementary material for: Exploring the potential of cell-free RNA and Pyramid Scene Parsing Network for early preeclampsia screening
Source: BMC Pregnancy Childbirth. 2025 Apr 14;25:445. doi: 10.1186/s12884-025-07503-5 (PMC11995606; doi:10.1186/s12884-025-07503-5)
Supplement: Supplementary file 3 — Supplementary Material 3. [file 12884_2025_7503_MOESM3_ESM.docx]

**Supplementary Table 3 Evaluation results on 13~20 gws data in multiple metric**

| **Method** | **MAE** | **Precision** | **Recall** | **AUC** | **F1 Score** |
| --- | --- | --- | --- | --- | --- |
| PSPNet | **0.055** | **0.923** | **0.971** | **0.8264** | **0.946** |
| MLP | 0.077 | 0.824 | 0.969 | 0.7349 | 0.898 |
| CNN | 0.0634 | 0.913 | 0.958 | 0.8054 | 0.913 |
